# Supplementary figures and images for: Jmjd1c is dispensable for healthy adult hematopoiesis and Jak2V617F-driven myeloproliferative disease initiation in mice
Source: PLoS One. 2020 Feb 4;15(2):e0228362. doi: 10.1371/journal.pone.0228362 (PMC6999878; doi:10.1371/journal.pone.0228362)

S1 Fig

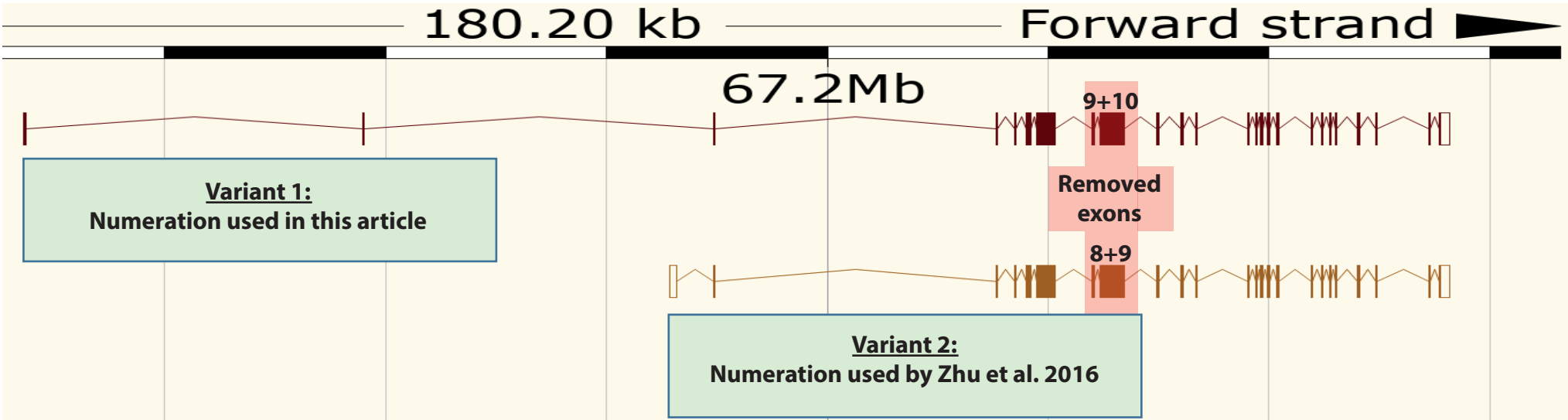

Supplement: S1 Fig — Exon numeration for protein coding variant 1 (Ensembl: ENSMUST00000174408.7, NCBI: NM_207221.2) and protein coding variant 2 (Ensembl: ENSMUST00000173689.7, NCBI: NM_001242396.1). This article uses the numeration of variant 1. Other articles might use variant 2 for exon numeration [3]. Exons removed in the d allele of Jmjd1c knockout mice are highlighted in red. This figure is based on the Ensembl genome browser. (PDF) [file pone.0228362.s001.pdf]

## S2 Fig

**A**

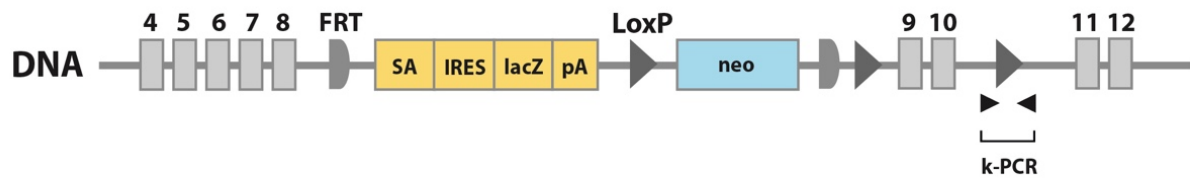

**B**

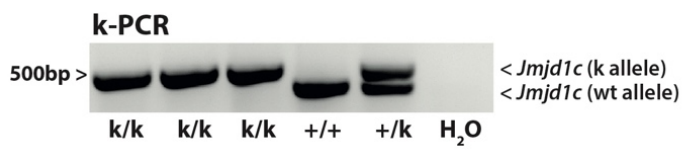

Supplement: S2 Fig — (A) Schematics of the knockout first approach (Jmjd1c-k allele). (B) Genotyping PCR with genomic DNA extracted from ear tissue. (PDF) [file pone.0228362.s002.pdf]

S3 Fig

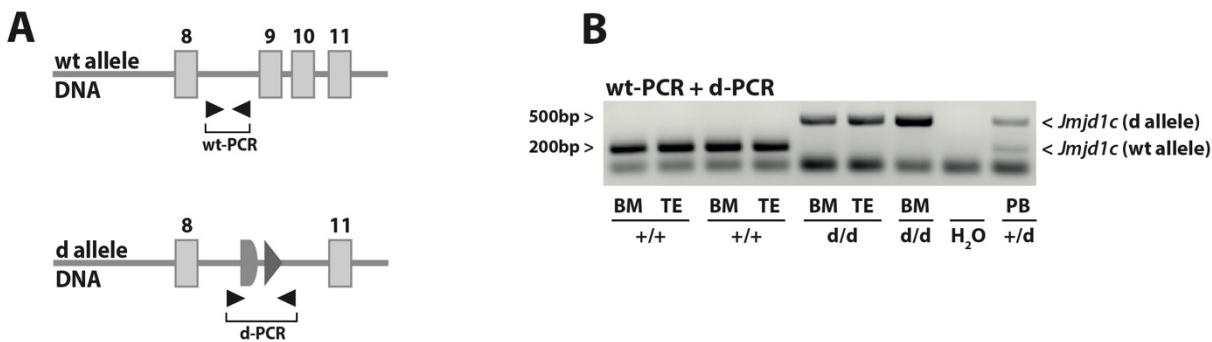

Supplement: S3 Fig — (A) Primer locations for genotyping PCRs. (B) Multiplex PCR for genotyping using material obtained from bone marrow (BM), testicular tissue (TE) or peripheral blood (PB). (PDF) [file pone.0228362.s003.pdf]

## S5 Fig

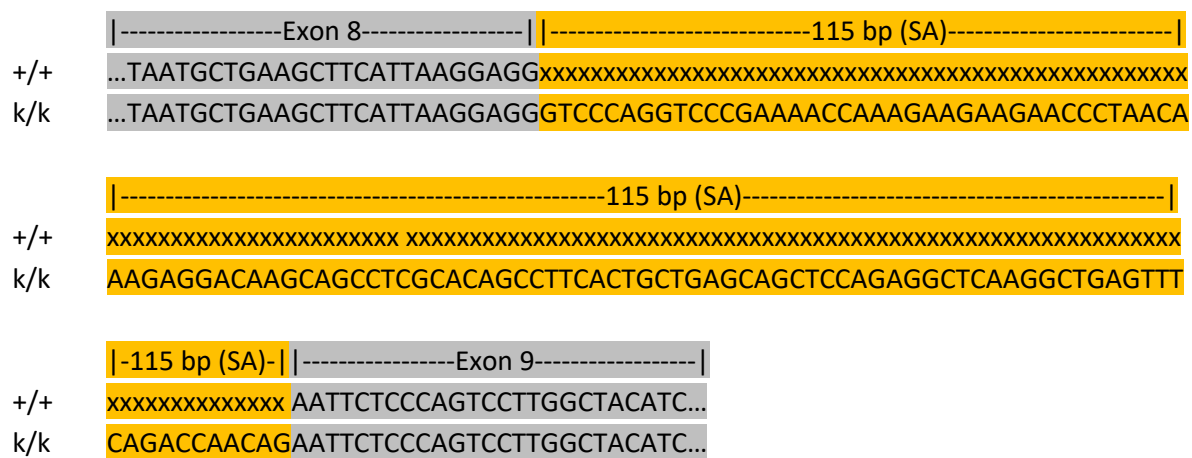

Supplement: S5 Fig — This supplemental figure relates to Fig 2B+2C. The last 25 bp of exon 8 and the first 25 bp of exon 9 are highlighted in grey. The 115 bp (SA) insert is highlighted in orange. (PDF) [file pone.0228362.s005.pdf]

**S6 Fig****A Kidney development**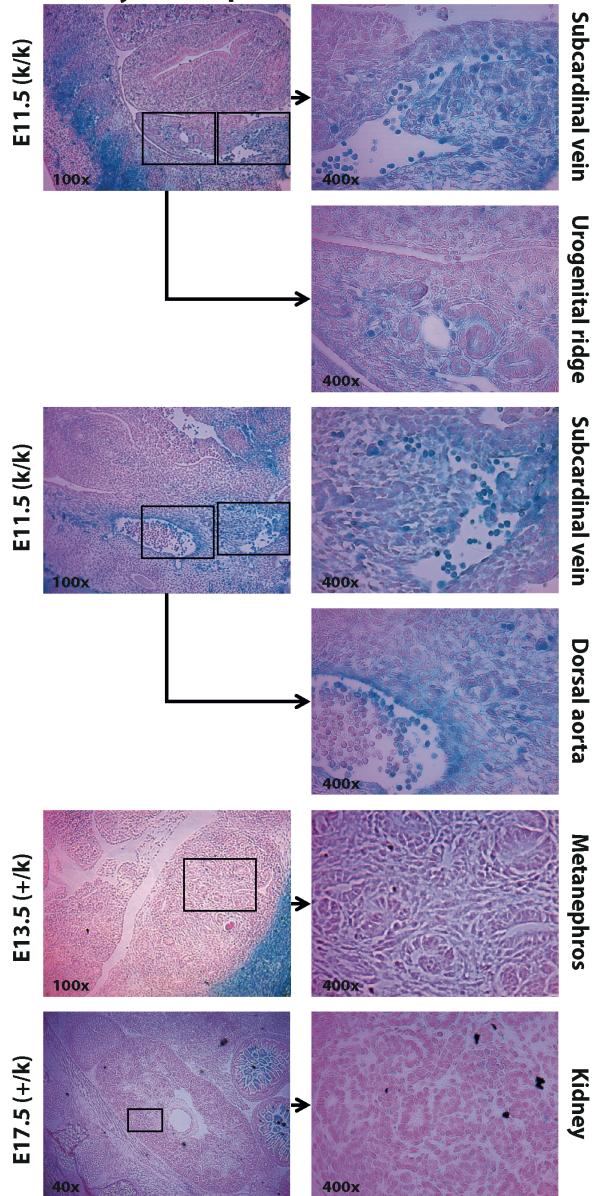**B Neurogenesis**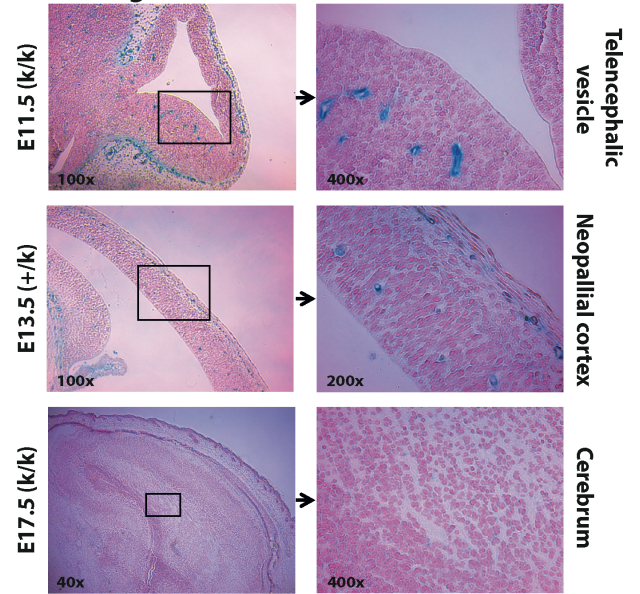**C Lung development**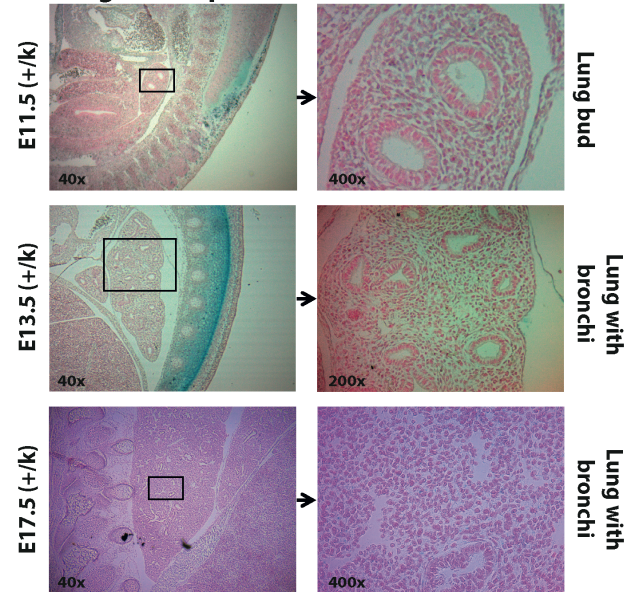**D Intestinal tract**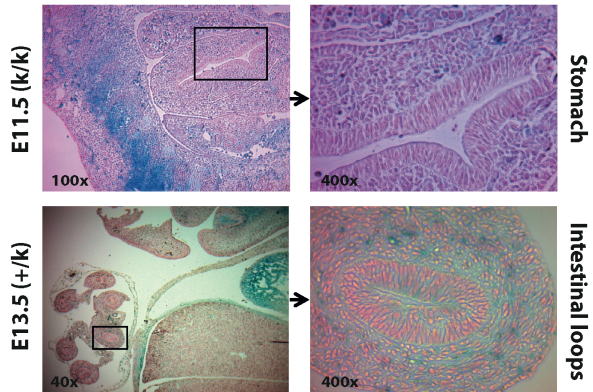**E**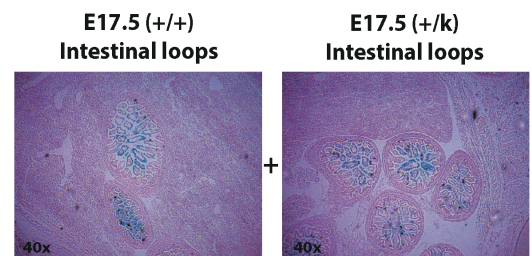

Supplement: S6 Fig — Sections showing beta-galactosidase signals during kidney development (A), neurogenesis (B), lung development (C) and development of the intestinal tract (D). (A-D) E11.5 and E13.5 whole mouse embryos were beta-galactosidase stained with subsequent formalin-fixation, paraffin embedding and sectioning, while E17.5 were first frozen followed by beta-galactosidase staining of the cryosections. All sections were counterstained with Nuclear Fast Red. (E) Background beta-galactosidase activity in the intestine of E17.5 embryos. (PDF) [file pone.0228362.s006.pdf]

S7 Fig

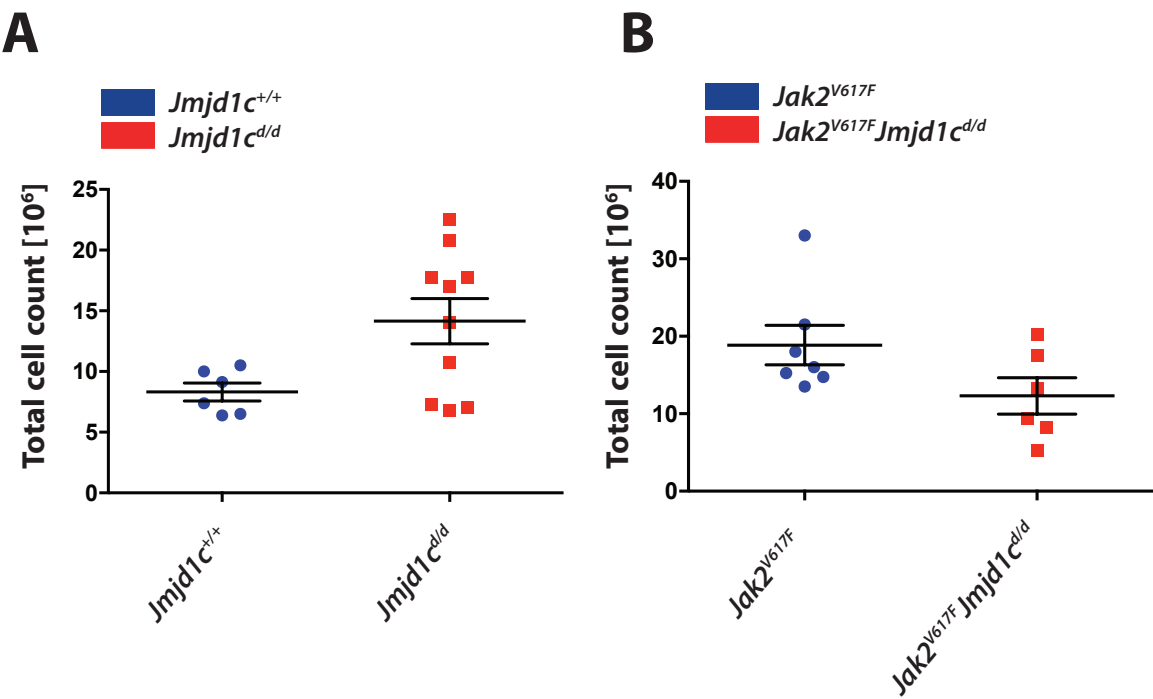

Supplement: S7 Fig — (A) Bone marrow cellularity in Jmjd1c+/+ and Jmjd1cd/d mice (n = 10 per genotype, lost data points are shown in S2 Table) after 40 weeks. The cell count of one femur was added to the cell count of one tibia. (B) Bone marrow cellularity in Jak2V617F and Jak2V617F Jmjd1cd/d mice (n = 6–7 per genotype) after 12 weeks. The cell count of one femur was added to the cell count of two tibiae. (A) and (B) Mann-Whitney U test was used for statistical calculations. (PDF) [file pone.0228362.s007.pdf]
