# Supplementary material for: Jmjd1c is dispensable for healthy adult hematopoiesis and Jak2V617F-driven myeloproliferative disease initiation in mice
Source: PLoS One. 2020 Feb 4;15(2):e0228362. doi: 10.1371/journal.pone.0228362 (PMC6999878; doi:10.1371/journal.pone.0228362)
Supplement: S4 Fig — (A-C) Full gel of picture shown in Fig 2C (A), Fig 3A (B+C). (B) Depicts wild type splicing. (C) Gel showing alternate splicing. (D+E) Uncropped wb presented in Fig 3B probed with an antibody against Jmjd1c (D) or β-actin (E). Lanes labeled with “unrelated experiments” have been previously published by us [1]. (F+G) Gel pictures relating to S2 Fig (F) and S3 Fig (G). (PDF) [file pone.0228362.s004.pdf]

S4 Fig

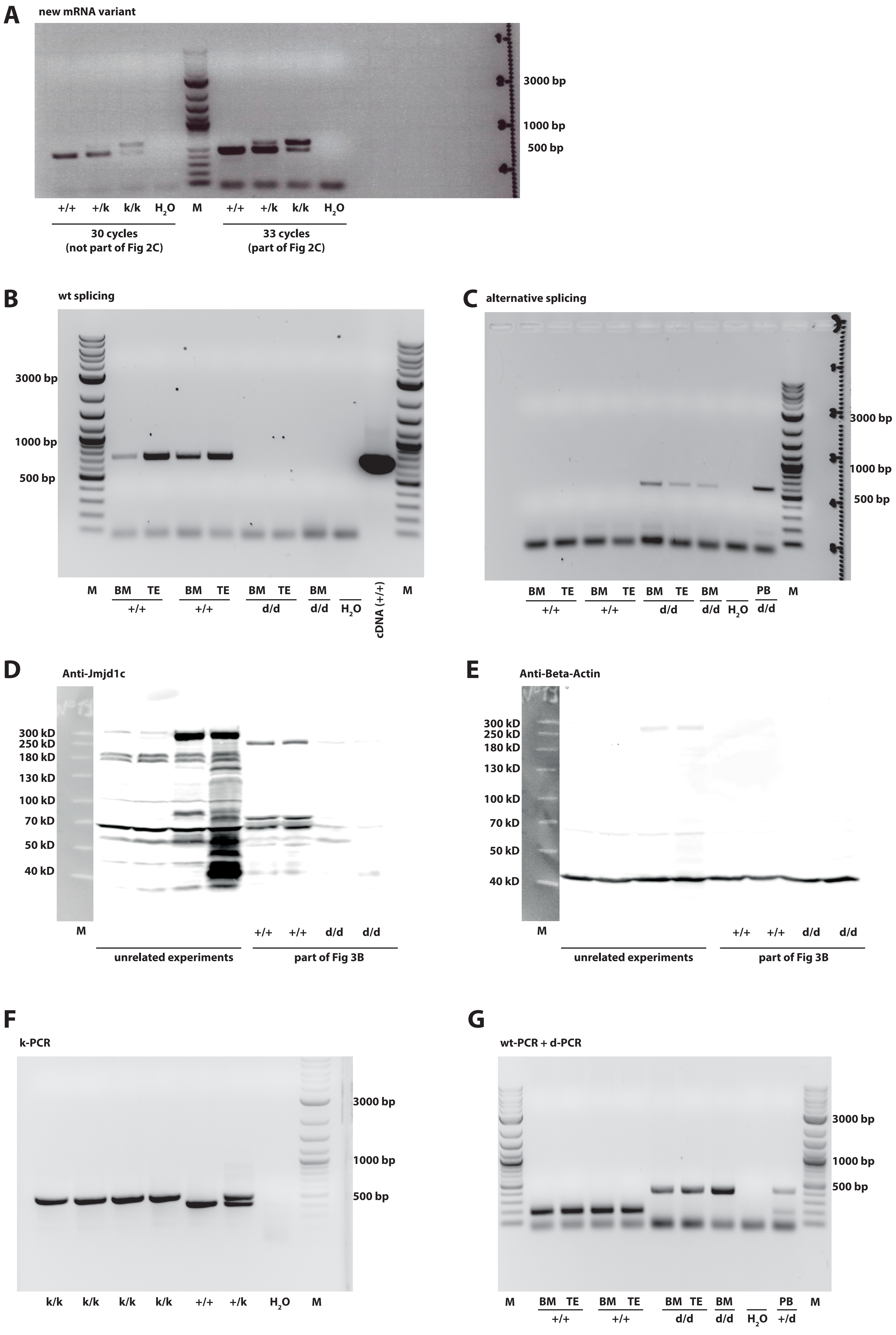

A
new mRNA variant
3000 bp
1000 bp
500 bp
+/+ +/k k/k H2O M +/+ +/k k/k H2O
30 cycles
(not part of Fig 2C)
33 cycles
(part of Fig 2C)
B
wt splicing
3000 bp
1000 bp
500 bp
M BM TE BM TE BM TE BM H2O cDNA (+/+) M
+/+ +/+ d/d d/d
C
alternative splicing
3000 bp
1000 bp
500 bp
BM TE BM TE BM TE BM H2O PB M
+/+ +/+ d/d d/d d/d
D
Anti-Jmjd1c
300 kD
250 kD
180 kD
130 kD
100 kD
70 kD
50 kD
40 kD
M +/+ +/+ d/d d/d
unrelated experiments
part of Fig 3B
E
Anti-Beta-Actin
300 kD
250 kD
180 kD
130 kD
100 kD
70 kD
50 kD
40 kD
M +/+ +/+ d/d d/d
unrelated experiments
part of Fig 3B
F
k-PCR
3000 bp
1000 bp
500 bp
k/k k/k k/k k/k +/+ +/k H2O M
G
wt-PCR + d-PCR
3000 bp
1000 bp
500 bp
M BM TE BM TE BM TE BM H2O PB M
+/+ +/+ d/d d/d +/d
