# Supplementary material for: Jmjd1c is dispensable for healthy adult hematopoiesis and Jak2V617F-driven myeloproliferative disease initiation in mice
Source: PLoS One. 2020 Feb 4;15(2):e0228362. doi: 10.1371/journal.pone.0228362 (PMC6999878; doi:10.1371/journal.pone.0228362)
Supplement: S1 Table — (DOCX) [file pone.0228362.s008.docx]

**S1 Table. Primer sequences.**

| Genotyping PCRs | | |
| --- | --- | --- |
| Name | forward primer (5’-->3’) | reverse primer (5’-->3’) |
| wt-PCR | GAAGTGATCTGGGAGTTGTGC | ATGCCCACGGACCTAATTTT |
| k-PCR | CAGGAGCGCCAAGGACAAAAG | AGACGGACCACATCACACTGCTC |
| d-PCR | AAGGCGCATAACGATACCAC | AGACGGACCACATCACACTGCTC |
| RT-PCRs | | |
| Name | forward primer (5’-->3’) | reverse primer (5’-->3’) |
| Exons 8/10 ^(a)^ | ACAGCACCAGTCACCTCATC | GCCTTGGACCCACAAAATGC |
| Exons 8/10 ^(b)^ | AACGCCAGGACCCCACTTACT | TGGACCCACAAAATGCCTATGC |
| Exons 8/11 | AACGCCAGGACCCCACTTACT | TTGATTCTTCCCCCTTCTTACTCC |

Legend to S1 Table. Sequences of oligonucleotides used in the RT-PCR (Exons 8/10), represented in Fig 1D (a) and Fig 2B (b).
