# Supplementary material for: Jmjd1c is dispensable for healthy adult hematopoiesis and Jak2V617F-driven myeloproliferative disease initiation in mice
Source: PLoS One. 2020 Feb 4;15(2):e0228362. doi: 10.1371/journal.pone.0228362 (PMC6999878; doi:10.1371/journal.pone.0228362)
Supplement: S2 Table — (DOCX) [file pone.0228362.s009.docx]

**S2 Table. Lost data points.**

| **Figure** | **Value** | **Time point** | **Genotype** | **Data points** | **Reason** |
| --- | --- | --- | --- | --- | --- |
| 4A | WBC | 24 weeks | *Jmjd1c^+/+^* | 2 out of 10 | ^(a)^ |
| 4A | WBC | 40 weeks | *Jmjd1c^+/+^* | 1 out of 10 | ^(b)^ |
| 4A | RBC | 40 weeks | *Jmjd1c^+/+^* | 1 out of 10 | ^(b)^ |
| 4A | PLT | 8 weeks | *Jmjd1c^d/d^* | 1 out of 10 | ^(c)^ |
| 4A | RETIC | 8 weeks | *Jmjd1c^+/+^* | 3 out of 10 | ^(d)^ |
| 4A | RETIC | 24 weeks | *Jmjd1c^+/+^* | 3 out of 10 | ^(d)^ |
| 4A | RETIC | 24 weeks | *Jmjd1c^d/d^* | 6 out of 10 | ^(d)^ |
| 4A | RETIC | 40 weeks | *Jmjd1c^+/+^* | 1 out of 10 | ^(b)^ |
| 4B | B220+ | 8 weeks | *Jmjd1c^d/d^* | 1 out of 10 | ^(e)^ |
| 4B | B220+ | 24 weeks | *Jmjd1c^d/d^* | 1 out of 10 | ^(f)^ |
| 4B | B220+ | 40 weeks | *Jmjd1c^+/+^* | 1 out of 10 | ^(b)^ |
| 4B | CD3+ | 8 weeks | *Jmjd1c^d/d^* | 3 out of 10 | ^(g)^ |
| 4B | CD3+ | 24 weeks | *Jmjd1c^d/d^* | 1 out of 10 | ^(f)^ |
| 4B | CD3+ | 40 weeks | *Jmjd1c^+/+^* | 1 out of 10 | ^(b)^ |
| 4B | Mac1/Gr1 | 8 weeks | *Jmjd1c^d/d^* | 3 out of 10 | ^(i)^ |
| 4B | Mac1/Gr1 | 24 weeks | *Jmjd1c^d/d^* | 1 out of 10 | ^(f)^ |
| 4C-E/4G | all values | 40 weeks | *Jmjd1c^+/+^* | 1 out of 10 | ^(b)^ |
| 4A-H | all values | all time points | *Jmjd1c^+/+^* | 1 out of 10 | ^(h)^ |
| 5J | MPP | 12 weeks | *Jak2^V617F^* | 1 out of 7 | ^(j)^ |
| S8A | total cells | 40 weeks | *Jmjd1c^+/+^* | 1 out of 10 | ^(b)^ |
| S8A | total cells | 40 weeks | *Jmjd1c^+/+^* | 1 out of 10 | ^(h)^ |
| S8A | total cells | 40 weeks | *Jmjd1c^+/+^* | 2 out of 10 | ^(k)^ |

Legend to S2 Table. **(a)** Result of a technical error in the measurement of the WBC on the ADVIA120. **(b)** Premature death before study end point. **(c)** Technical error in the measurement of the PLTs. **(d)** Defect of the RETIC channel of the ADVIA120. **(e)** Shortage in the supply of B220 antibody. **(f)** We lost all flow data points because the cell count was too low for this mouse at this time point. **(g)** Shortage in the supply of CD3e antibody. **(h)** The genotype of this mouse could not reliably be verified and while it clearly grouped with *Jmjd1^+/+^* mice in values, all values were excluded henceforth and the mouse was sacrificed. **(i)** The samples were measured with wrong settings of the red laser, a recurrent error reported to the institute by the manager of the core facility. Therefore, we had to exclude the following values: Mac1+/Gr1+ (1,38 %, 2,61 %, 1,77 %) and Mac1+/Gr1- (9,4 %, 12,4 %, 15,7 %). **(j)** The MPP value of one mouse in the *Jak2^V617F^*-group (0,000098 %) deviates more than 100-fold from the mean (0,022 %) and known reference values obtained in the lab. Therefore, the value was excluded as an outlier. **(k)** In two mice of the *Jmjd1c^+/+^*-group, the total cell count was only determined in one femur (8,75 and 9,75 x 10^6^ cells). Therefore, these data points are not comparable to the other values (cell count for one femur added to the cell count of one tibia).
